# *SiNCED1*, a 9-cis-epoxycarotenoid dioxygenase gene in *Setaria italica*, is involved in drought tolerance and seed germination in transgenic Arabidopsis

**DOI:** 10.3389/fpls.2023.1121809

**Published:** 2023-03-09

**Authors:** Yuan Huang, Yang Jiao, Sha Yang, Dandan Mao, Feng Wang, Liangbi Chen, Manzhong Liang

**Affiliations:** ^1^ College of Pharmacy, Guizhou University of Traditional Chinese Medicine, Guiyang, China; ^2^ College of Life Science, Hunan Normal University, Changsha, China; ^3^ Hunan Province Key Laboratory of Crop Sterile Germplasm Resource Innovation and Application, College of Life Science, Hunan Normal University, Changsha, China

**Keywords:** foxtail millet, abscisic acid, SiNCED1, drought stress, seed germination

## Abstract

Foxtail millet (*Setaria italica* L.) is a vital cereal food crop with promising development and utilization potential because of its outstanding ability to resist drought stress. However, the molecular mechanisms underlying its drought stress resistance remain unclear. In this study, we aimed to elucidate the molecular function of a 9-cis-epoxycarotenoid dioxygenase gene, *SiNCED1*, in the drought stress response of foxtail millet. Expression pattern analysis showed that *SiNCED1* expression was significantly induced by abscisic acid (ABA), osmotic stress, and salt stress. Furthermore, ectopic overexpression of *SiNCED1* could enhance drought stress resistance by elevating endogenous ABA levels and promoting stomatal closure. Transcript analysis indicated that *SiNCED1* modulated ABA-related stress responsive gene expression. In addition, we found that ectopic expression of *SiNCED1* delayed seed germination under normal and abiotic stress conditions. Taken together, our results show that *SiNCED1* plays a positive role in the drought tolerance and seed dormancy of foxtail millet by modulating ABA biosynthesis. In conclusion, this study revealed that *SiNCED1* is an important candidate gene for the improvement of drought stress tolerance in foxtail millet and could be beneficial in the breeding and investigation of drought tolerance in other agronomic crops.

## 1 Introduction

Foxtail millet (*Setaria italica* L.) is an important cereal food crop that has been cultivated for over 8000 years (Zhang et al., 2012). This species is an ideal model plant because of its short life cycle, self-pollination, small diploid genome, prolific seed production, and small adult stature (Doust et al., 2009). Moreover, foxtail millet is a promising crop for the study of drought stress because of its outstanding ability to resist it (Barton et al., 2009). However, the genes underlying the foxtail millet drought stress response have not been studied extensively to date.

Abscisic acid (ABA) is an essential phytohormone that regulates plant development and modulates abiotic stress tolerance. The main functions of ABA in plant development include dormancy, the delaying of seed germination, and induction of stomatal closure (Yang et al., 2022). For example, ABA plays a critical role in the protection of seed dormancy and inhibition of seed germination through ABA metabolism, which is modulated by regulatory factors, the environment, and natural variations of ABA metabolism signal pathway related genes (Sano and Marion-Poll, 2021). Moreover, ABA promotes stomatal closure in a dual way by increasing its concentration in guard cells and decreasing water permeability in leaf vascular tissues (Pantin et al., 2013). During exposure to abiotic stresses, such as drought, salinity, heat, and cold, ABA can act as a dominant hormone to regulate abiotic stress signaling pathways. For instance, ABA promotes desiccation-tolerance by controlling stomatal closure, enabling plants to adapt to water stress (Shang et al., 2020). In addition, ABA participates in plant development, and abiotic-stress tolerance depends on ABA metabolism (biosynthesis and catabolism), perception, the core signaling pathway, and factors that trigger ABA-mediated transcription (Nambara et al., 2010).

Endogenous ABA levels in plants are positively regulated by ABA biosynthesis (Ng et al., 2014). ABA biosynthesis occurs in two sites, starting in the plastids and ending in the cytosol (Sah et al., 2016). ABA biosynthesis has two pathways, but in higher plants, ABA is synthesized *via* an indirect pathway, also called the mevalonic acid-independent pathway. Several enzymes that originate from the catalysis of carotenoid precursors participate in this pathway (Xu et al., 2013). To date, almost all biosynthetic genes have been identified through the isolation of nutritionally deficient mutants or the creation of specific gene-editing mutants (Eiji and Annie, 2005; Miao et al., 2013), including zeaxanthin epoxidase (*ZEP*), 9-cis-epoxycarotenoid dioxygenase (*NCED*), and abscisic aldehyde oxidase (*AAO*) (Andrew and Roger, 1992; Finkelstein, 2013). The NCED enzyme regulates the key limiting step of ABA biosynthesis by catalyzing the cleavage of 9-cis-violaxanthin or 9-cis-neoxanthin to xanthoxin (C15) (Ye et al., 2012).

The first *NCED* gene, *VP14*, was cloned and identified in maize (*Zea mays* L.) (Schwartz et al., 1997). Subsequently, *NCED* genes have been isolated and studied from multiple plant species in both dicotyledons and monocotyledons, such as Arabidopsis (*Arabidopsis thaliana* (L.) Heynh.) (Sato et al., 2018), peaches (*Prunus persica* L.) (Wang et al., 2021), cotton (*Gossypium hirsutum* L.) (Li et al., 2021), *Brassica napus* L. (Xu and Cai, 2017), tomatoes (*Solanum lycopersicum* L.) (Sun et al., 2012), rice (*Oryza sativa* L.) (Ye et al., 2011), wheat (*Triticum aestivum* L.) (Lang et al., 2021), and *Phaius tankervilliae* (Banks ex L’Hér.) Blume (Lee et al., 2018). The rice *NCED* gene family consists of five members (Ye et al., 2011), with the following functions: *OsNCED1*, the overexpression of which can improve the heat tolerance of rice by enhancing its antioxidant capacity (Zhou et al., 2022); *OsNCED3* and *OsNCED5*, which mediate multi-abiotic stress tolerance and leaf senescence by regulating endogenous ABA accumulation in rice (Huang et al., 2018; Huang et al., 2019); and *OsNCED3*, *OsNCED4*, and *OsNCED5*, whose ectopic expression in Arabidopsis can alter plant size and leaf morphology, delay seed germination, and advanced drought stress tolerance through the regulation of endogenous ABA content (Hwang et al., 2010; Hwang et al., 2018; Huang et al., 2019). In Arabidopsis, five *NCED* genes have been identified (Tan et al., 2003), which exhibit diverse expression patterns and share redundant functions. *AtNCED6* cooperates with *AtNCED9* to regulate seed germination and dormancy by controlling ABA levels in seeds. The transcription of *AtNCED3* and *AtNCED5* can be strongly induced by drought stress, which results in increased endogenous ABA levels and improved water stress tolerance (Hao et al., 2009; Frey et al., 2012). Two *NCED* isozymes, *PpNCED1* and *PpNCED5*, which have been identified in peach fruits, control fruit ripening and senescence by regulating ABA biosynthesis (Wang et al., 2021).

The above studies have shown that increasing the transcript levels of *NCED* could promote ABA biosynthesis and enhance ABA accumulation in plants, which play important roles during the various phases of the plant life cycle, including seed development and dormancy, and in plant responses to variable environmental stress (Koshiba, 2002). However, the functions of ABA biosynthesis genes for *NCED* in foxtail millet are still unknown. In this study, we cloned four *NCED* genes from the *Setaria italica* cultivated variety Jigu 42. The expression pattern and ectopic expression of *SiNCED1* in Arabidopsis was investigated. *SiNCED1* was strongly induced by ABA, osmotic stress, and salt stress. Furthermore, *SiNCED1* overexpression increased endogenous ABA contents to enhance drought stress tolerance and delay seed dormancy. Our findings indicate that *SiNCED1* can be an important candidate gene to improve drought stress tolerance and keep seed dormancy in foxtail millet and other agronomic crops.

## 2 Materials and methods

### 2.1 Plant growth conditions and treatment

Foxtail millet (*S. italica*, cultivar Jigu 42) seeds were germinated in distilled water for 3 d at 28°C. The seedlings were grown in Hoagland’s culture solution (Li and Cheng, 2015) and cultured in growth chambers at 28°C under a 16 h light/8 h dark cycle. For abiotic stress, two-week-old seedlings were subjected to various stress treatments by placing them in a solution with a final concentration of 20% PEG6000, 150 mM NaCl, and 100 μM ABA. All treatments were repeated more than three times. The shoots and roots were harvested for RNA extraction.

*A. thaliana* (Columbia-0 ecotype; Col) was used in this study. The seeds were surface-sterilized with 75% (v/v) ethanol and sown on either a 1/2 MS medium or soil at 22°C under a 16 h light/8 h dark cycle (Lu et al., 2017). To analyze the seed-germination rate, sterilized seeds were sown on a 1/2 MS medium with 150 mM NaCl, 275 mM mannitol, and 6% glucose. The seed germination rate were calculated according to opened cotyledons. For drought stress treatment, the Arabidopsis transgenic lines and Col were grown in soil for 25 d under the same growth conditions mentioned above; after which, water was withheld for 30 d, followed by watering for 7 d.

### 2.2 Phylogenetic relationship and multiple alignment analysis

The NCED protein sequences from four species, *A. thaliana*, *Oryza sativa*, *Zea mays*, and *Setaria italica*, were used for phylogenetic analysis. Phylogenetic tree analysis was performed using MEGA 7 software utilizing the maximum-likelihood method. Multiple sequence alignment was used to construct the phylogenetic tree in MEGA7 with the following parameter settings: Jones-Taylor-Thornton, pairwise deletion, and 1000 bootstrap replications.

The protein sequences of *NCED* genes used for multiple alignment were downloaded from the NCBI database (http://plants.ensembl.org/index.html). The multiple alignment was conducted using Bioedit software, applying ClustalW with the following parameters: full multiple alignment, number of bootstraps 1000, output clustal format with clustal consensus sequence generation.

### 2.3 Generation of *SiNCED1*-overexpressing lines

The *SiNCED1* open reading frame (ORF) was amplified through PCR using pHB-NCED1-F/R primers (**Supplementary Table S1**) and then inserted into the pHB binary vector with HindIII and XbaI restriction sites. The recombinant construct, 2×35S::*SiNCED1*, was transformed into Arabidopsis using the floral dip transformation method. The transgenic lines were planted on MS medium containing 50 μg hygromycin for 7 d. Homozygous lines with 100% resistance to hygromycin were selected and confirmed through semi-quantitative-PCR. Two genetically stable transgenic lines (OE3 and OE5) with high transcript levels of *SiNCED1* were selected for further analysis.

### 2.4 Quantitative real-time PCR analysis

Total RNA extraction was carried out for all samples using Trizol (Invitrogen, Shanghai, China) and was converted to cDNA using the Synthesis Kit (Thermo Fisher Scientific, USA) according to the manufacturer’s instructions. Subsequently, quantitative real-time PCR (RT-qPCR) was performed using the SYBR Premix Ex Taq kit (Takara, Dalian, China) on an Applied Biosystems Quant Studio 5 (Thermo Fisher Scientific). Relative changes in gene expression levels were quantitated based on three biological replicates using the 2^-ΔΔCt^ method (Livak and Schmittgen, 2001). Each experiment included three technical replicates. The primer sequences used for RT-qPCR are listed in **Supplementary Table S1**.

### 2.5 Analysis of water loss rate, proline, electrolyte leakage and ABA content

The water loss rate was measured as described previously (Huang et al., 2018). The Arabidopsis rosette leaves of 25-day-old Col and *SiNCED1*-overexpressing lines were detached and placed in dishes with constant conditions. Fresh weight was measured at set times (0, 10, 20, 30, 40, 50, 60, 90,120, 150, and 180 min). The proportion of the initial fresh weight represented the water loss rate.

The proline content was measured according to previously described methods (Huang et al., 2018). Approximately 300 mg fresh leaf samples were homogenized in 3 mL of 3% aqueous sulfosalicylic acid and centrifuged at 4000 × *g* for 10 min. Then, 1 mL of supernatant was taken out and mixed with 1 mL of acid ninhydrin and 1 mL of glacial acetic acid in a new tube and then incubated in boiling water bath for 40 min. For every reaction, 2 mL toluene was added and oscillated for 30 min at room temperature. The proline content was measured using a spectrophotometer at 520 nm.

Relative electrolyte leakage was measured as previously described (Lou et al., 2017). The same number of detached leaf samples were placed in 20 mL of deionized water and then in a vacuum pump for 30 min. Samples were then sealed and placed in a 25 °C incubator for 6 h. The water conductance was measured using a conductivity meter.

ABA content was measured as previously described (Huang et al., 2019). Arabidopsis rosette leaves (300 mg) were collected from 25-day-old Col and *SiNCED1*-overexpressing lines and ground in liquid nitrogen. The internal standard was [^2^H_6_], and ABA content was quantified using UPLC–MS/MS with three independent biological replicates.

### 2.6 Measurement of stomatal aperture

Stomatal apertures were analyzed using *Arabidopsis* leaf epidermis samples under normal or drought stress conditions. Stomata were observed and photographed under a fluorescence microscope (BX51, Olympus, Japan). The size of stomatal apertures was measured using Image J software and a digital ruler. Three independent experiments were performed, which obtained similar results. Each experiment included at least 30 stomata from each epidermal sample.

### 2.7 Statistical analysis

All experiments were repeated at least three times for each treatment. Data were analyzed using SPSS software (version 13.0). Differences were considered statistically significant at *P <* 0.05, as determined using ANOVA analysis and Tukey’s multiple comparisons test. All data are shown as mean ± SD from the three independent experiments.

## 3 Results

### 3.1 Molecular characterization of *NCED* in foxtail millet

**Figure 1:**
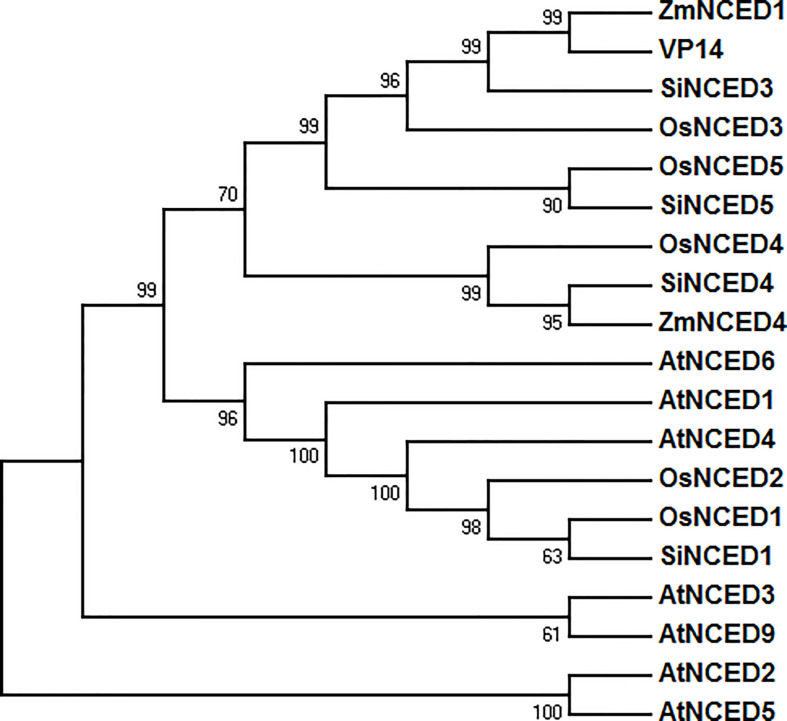
Phylogenetic tree analysis based on the amino acid sequences of SiNCED1 and other NCED from other plants. Nineteen amino acid sequences from four species were selected and analyzed by MEGA 7.

**Figure 2:**
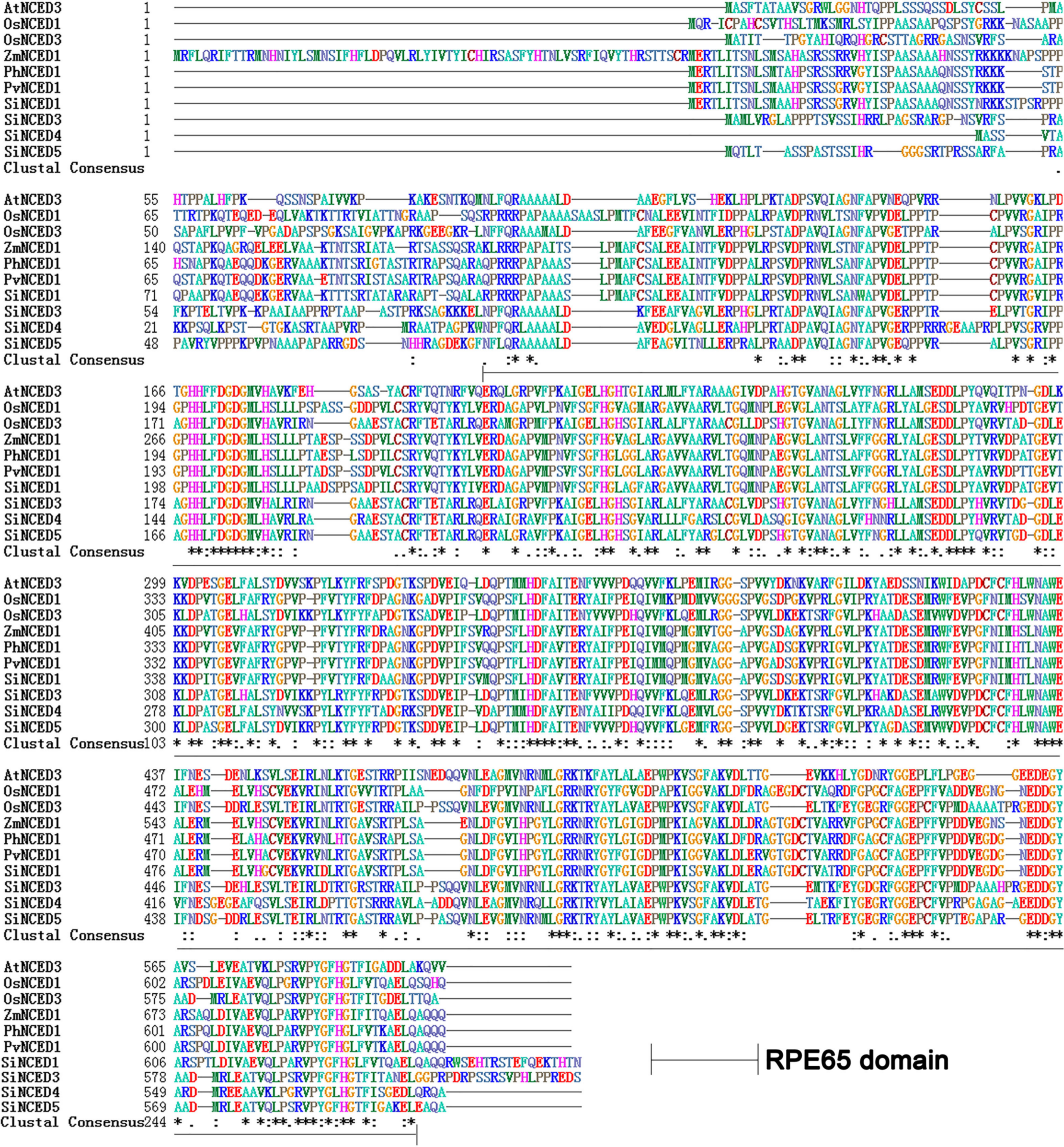
Multiple alignment of the deduced protein sequence of SiNCED1 and NCED proteins from other plants. Ten protein sequences from six species were selected and analyzed by Bioedit.

Four 9-*cis*-epoxycarotenoid dioxygenase genes were found in the foxtail millet genome database (NCBI GenBank) and cloned (**Supplementary Table S2**). These genes were designated as *SiNCED1* (LOC101783411), *SiNCED3* (LOC101778945), *SiNCED4* (LOC101766978), and *SiNCED5* (LOC101770668). Among these, *SiNCED1* was further investigated because of its promoter, which may have a drought related cis-element, as assessed through plantCARE prediction analysis (**Supplementary Table S3**). The full-length *SiNCED1* CDS has a 1980 bp ORF, encoding 660 amino acids. To understand the phylogenetic relationship of SiNCED protein, a phylogenetic tree was constructed among SiNCED1, other SiNCEDs, and NCED proteins derived from Arabidopsis, rice, and maize. Based on the analysis, SiNCED proteins were clustered into two clades (**Figure 1**). SiNCED1 was separately clustered into a clade, while the other three SiNCEDs were aggregated into another clade. Alignment of the deduced protein sequences showed that SiNCED1 contains a large number of conserved amino acid residues in the NCED conserved domain RPE65, when compared with other NCED proteins (**Figure 2**). Therefore, SiNCED1 is homologous with NCED proteins from other species.

### 3.2 Expression pattern analysis of *SiNCED1* in multiple tissues and under ABA or abiotic stresses treatments

**Figure 3:**
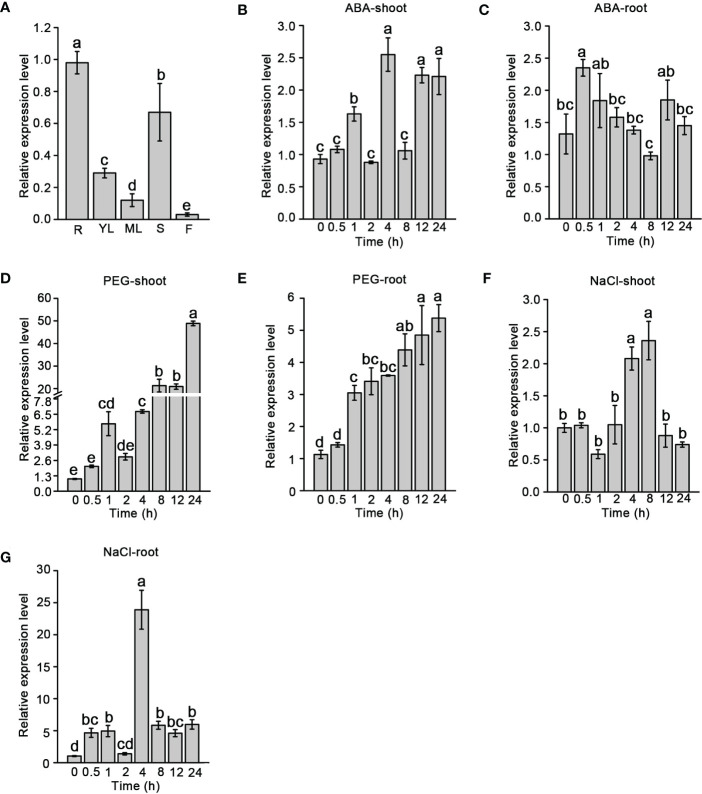
*SiNCED1* gene expression pattern. **(A)** *SiNCED1* gene expression in different organs. R(root), YL(young leaf), ML(mature leaf), S(stem), F(flower). **(B–G)** The three-leaf stage seedlings were submitted to ABA and 20% PEG, 150 mM NaCl treatment. **(B, C)** Expression of *SiNCED1* under ABA treatment; **(D, E)** Expression of *SiNCED1* under 20% PEG stress; **(F, G)** Expression of *SiNCED1* under 150 mM NaCl stress. The shoot and root samples were collected for *SiNCED1* expression analysis. Data are the mean ± SD for three replicates. Different letters represent significant differences at *P*<0.05 (ANOVA analysis, Tukey’s multiple comparisons test*)*.

The temporal and spatial expression patterns of *SiNCED1* were observed through RT-qPCR. *SiNCED1* was constitutively expressed in the roots, young leaves, mature leaves, stems, and flowers but highly expressed in the roots, following by the stems, young leaves, mature leaves and flowers (**Figure 3A**).

To investigate the response of *SiNCED1* transcript levels under ABA, 20% PEG, and 150 mM NaCl treatments, RT-qPCR was performed to analyzed *SiNCED1* expression in the shoots and roots of seedlings. Compared with the normal conditions, *SiNCED1* was strongly induced in the shoots and roots of seedlings that underwent ABA treatment. We found that the expression levels of *SiNCED1* reached their peak at 4 h in the shoots but 0.5 h in the roots (**Figures 3B, C** **)**. In roots and shoots that had undergone PEG treatment, the expression of *SiNCED1* was also induced, reaching their highest levels at 24 h in the shoots but 12 h in the roots (**Figures 3D, E** **)**. Under high salt treatment, the level of *SiNCED1* showed a trend of first increasing and then decreasing in the shoots and roots and reached its highest level in both at 4 h (**Figures 3F, G** **)**. These results suggest that *SiNCED1* plays a role in the tolerance of foxtail millet to multiple types of abiotic stress.

### 3.3 *SiNCED1* confers drought stress tolerance

**Figure 4:**
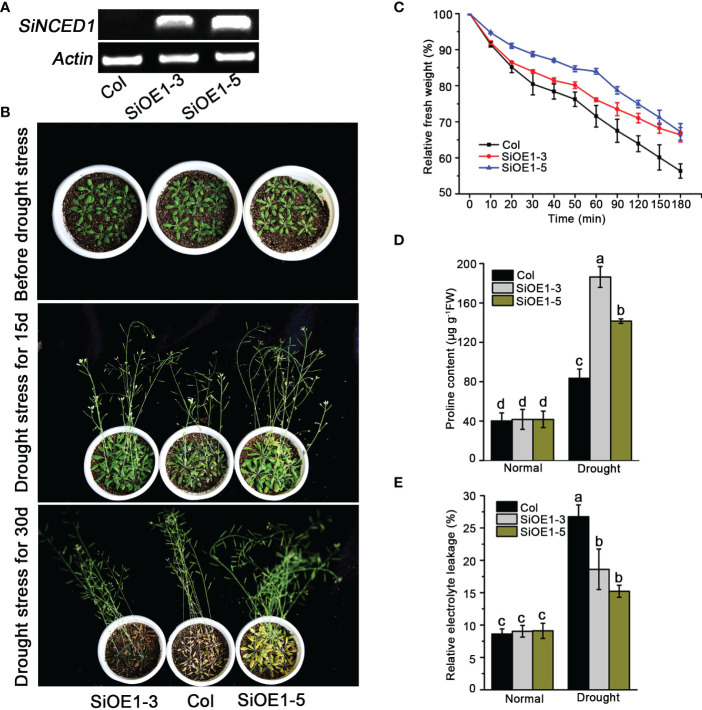
Drought stress tolerance of *SiNCED1* overexpression plants. **(A)** Semi-quantitative RT-PCR detection of *SiNCED1* expression in Col and SiOE1-3, SiOE1-5. **(B)** The phenotype of *SiNCED1*-overexpressing plants and Col seedlings under drought stress. **(C)** Measurement of water loss rate in Col and *siNCED1-*overexpressing seedlings. **(D)** Proline content determination after drought stress treatment **(E)** Relative electrolyte leakage determination after drought stress treatment. Data shown are mean ± SD from three independent replicates. Values are the mean ± SD (*n* = 6 plants). Similar results were obtained from three independent replicates. Different letters represent significant differences at *P*<0.05 (ANOVA analysis, Tukey’s multiple comparisons test*)*.

The heterogeneous expression of *SiNCED1* in Arabidopsis was investigated to determine the function of *SiNCED1*. Semi-quantitative PCR analysis showed that *SiNCED1* expression was only detected in *SiNCED1*-overexpressing lines (SiOE1-3 and SiOE1-5) but not in Col (**Figure 4A**). Subsequently, *SiNCED1*-overexpressing plants and Col were analyzed to identify the ability of drought stress tolerance. The *SiNCED1*-overexpressing plants and Col were grown in soil for 25 d under well-watered conditions, and water-stress conditions (water withheld) for 15 d and 30 d. When water was withheld for 30d, almost all Col plants withered, but *SiNCED1*-overexpressing lines did not show any wilted leaves (**Figure 4B**).

Given that the altered physiological mechanism was possibly responsible for drought stress adaptation, we examined the water loss rate, proline content, and relative electrolyte leakage in detached Arabidopsis rosette leaves. We observed that Col rosette leaves lost water faster than those from the *SiNCED1*-overexpressing lines (**Figure 4C**). Meanwhile, the proline content and relative electrolyte leakage were not substantially different between Col plants and *SiNCED1*-overexpressing lines under normal conditions. However, *SiNCED1*-overexpressing lines had higher proline content and lower relative electrolyte leakage than Col plants under drought stress (**Figures 4D, E** **)**. These results indicate that *SiNCED1* could increase drought stress tolerance in foxtail millet.

**Figure 5:**
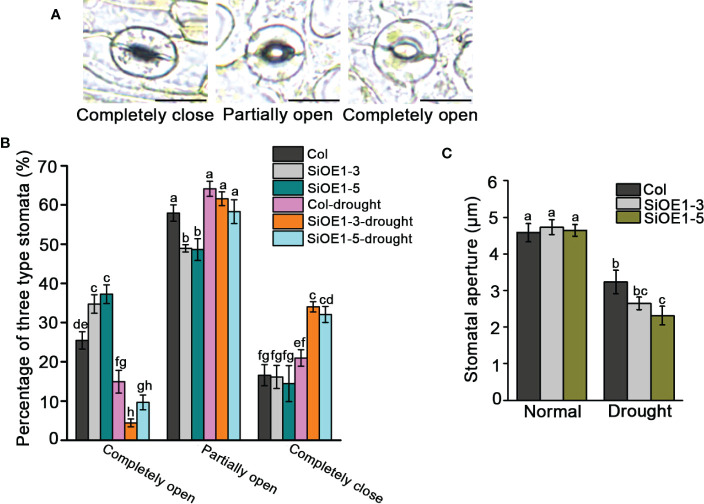
Stomata closure of *SiNCED1*-overexpressing plants under drought stress. **(A)** Images of three levels of stomatal apertures, Bars = 10 μm. **(B)** The percentage of three levels of stomatal apertures in *SiNCED1*-overexpressing plants and Col were calculated under normal and drought stress condition. **(C)** Comparison of stomatal apertures in *SiNCED1*-overexpressing plant and Col under normal and drought stress condition. Data shown are mean ± SD from there independent replicate. Each experiment includes at least 30 stomata from each epidermal peel. Different letters represent significant differences at *P*<0.05 (ANOVA analysis, Tukey’s multiple comparisons test*)*.

We also investigated stomatal aperture to further assess the drought stress tolerance in Col and *SiNCED1*-overexpressing plants. Stomatal apertures were classified into three categories: completely open, partially open, and completely closed (**Figure 5A**). Under normal conditions, the percentage of completely open stomata in *SiNCED1*-overexpressing plants was slightly higher than that in Col plants, but the percentage of completely closed and partially open stomata showed no difference between *SiNCED1*-overexpressing and Col plants (**Figure 5B**). However, under drought stress conditions, 34% and 32.05% of stomata were completely closed in *SiNCED1*-overexpressing plants (SiOE1-3 and SiOE1-5, respectively), whereas only 20.97% were completely closed in Col plants. Furthermore, only 4.43% and 9.65% of stomata were completely open in *SiNCED1*-overexpressing plants (SiOE1-3 and SiOE1-5, respectively), whereas 14.92% were completely open in Col. Additionally, 64.11% of stomata were partially open in Col, and no marked differences were observed compared with 61.57% and 58.3% of partially open stomata in *SiNCED1*-overexpressing plants (SiOE1-3 and SiOE1-5, respectively) (**Figure 5B**). Consistent with these results, the stomatal apertures did not differ between Col and *SiNCED1*-overexpressing plants under normal conditions. However, the stomatal apertures of *SiNCED1*-overexpressing plants (SiOE1-3, SiOE1-5) were significantly smaller than those of Col plants under drought conditions (**Figure 5C**). These results indicate that *SiNCED1* may reduce stomatal apertures to inhibit water loss under drought stress conditions.

### 3.4 *SiNCED1* increases ABA accumulation and ABA-related stress-responsive gene expression under drought stress

**Figure 6:**
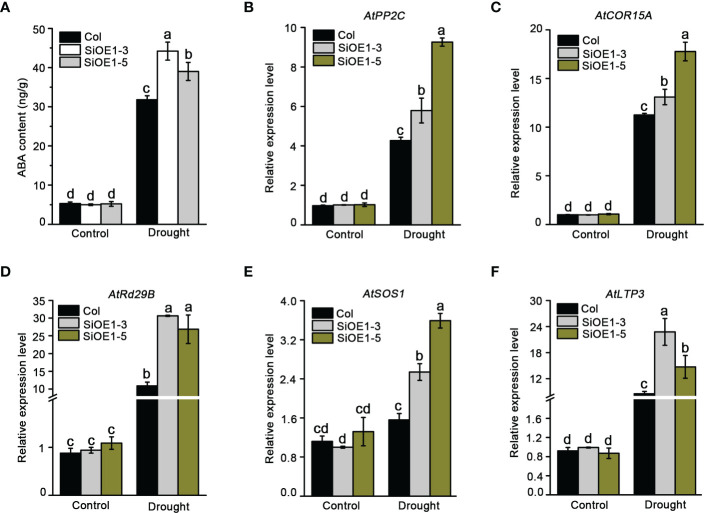
ABA content and abiotic stress related genes expression. **(A)** The ABA content in seedlings of Col and *SiNCED1*-overexpressing plants under drought stress. **(B–F)** The expression of ABA related stress responsive genes in Col and *SiNCED1*-overexpressing plants under drought stress for 7 d. Data shown are mean ± SD from there independent replicate. Different letters represent significant differences at *P*<0.05 (ANOVA analysis, Tukey’s multiple comparisons test*)*.

Stomata movement acts as an important mechanism for plant resistance to drought stress through an ABA-dependent pathway. Moreover, NCED is a critical rate-limiting enzyme in ABA biosynthesis. To understand whether endogenous ABA is involved in altering drought stress tolerance, ABA levels were measured in Col and *SiNCED1*-overexpressing plants before and after drought stress treatment. We found that under normal conditions, ABA levels were not different between Col and *SiNCED1*-overexpressing plants (**Figure 6A**). However, ABA levels rapidly increased under drought stress condition in both Col and *SiNCED1*-overexpressing plants, and the ABA levels in *SiNCED1*-overexpressing plants (SiOE1-3, 44.2 ng/g; SiOE1-5, 39 ng/g) were much higher than those in Col plants (31.8 ng/g) (**Figure 6A**). Therefore, *SiNCED1* increases drought stress tolerance by promoting endogenous ABA biosynthesis in plants.

We further determined the transcript levels of ABA-related stress-responsive genes under normal and drought stress conditions. Consistent with the changes in ABA content, the expression of *AtPP2C*, *AtCOR15A*, *AtRd29B*, *AtSOS1*, and *AtLTP3* did not differ between Col and *SiNCED1*-overexpressing plants under normal conditions. However, the expression levels of these genes were noticeably higher in *SiNCED1*-overexpressing plants than in Col plants (**Figures 6B–F**). These results suggest that *SiNCED1* up-regulated ABA-related stress-responsive genes under drought stress conditions.

### 3.5 Delayed seed germination of *SiNCED1-*overexpressing Arabidopsis

**Figure 7:**
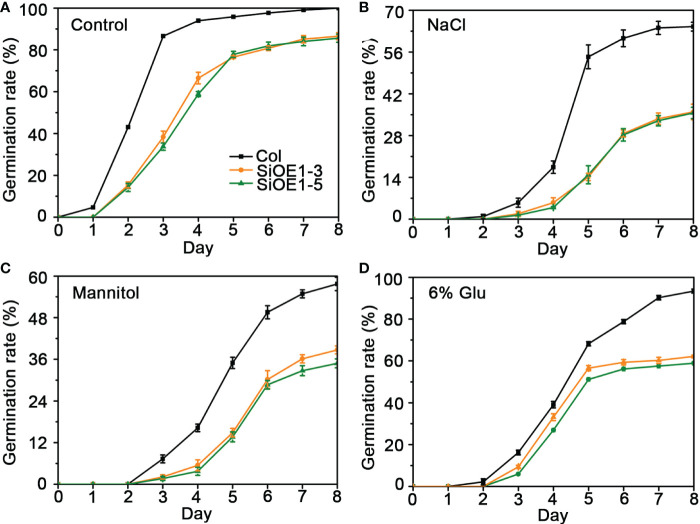
Germination rate of Col and *SiNCED1* transgenic seeds grown in MS, Mannitol, NaCl and Glucose media. **(A–D)** The germination rate of Col and *SiNCED1* transgenic seeds grown in MS media contain 150 mM NaCl, 275 mM Mannitol, or 6% Glucose. Data shown are mean ± SD from three independent replicates.

**Figure 8:**
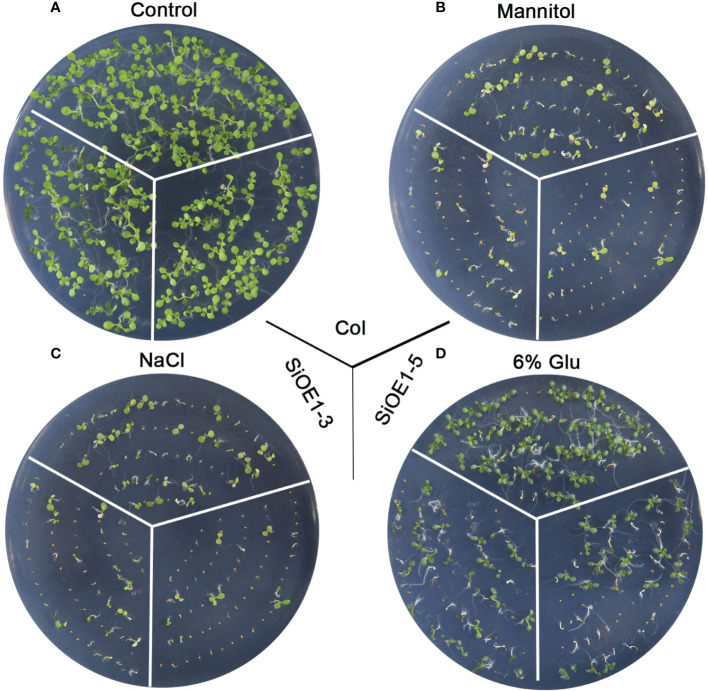
Phenotypic analysis of *SiNCED1* transgenic plants under MS, mannitol, NaCl and Glucose. **(A–D)** The phenotype of Col and *SiNCED1* transgenic seeds grown in MS (control), MS contain 275 mM Mannitol, 150 mM NaCl or 6% Glucose, respectively for 7 d.

The above results show that *SiNCED1* regulates endogenous ABA biosynthesis. Since ABA plays pivotal roles in seed dormancy and germination, the seed germination rate of Col and *SiNCED1*-overexpressing lines were determined. The results showed that the seed germination rate of *SiNCED1*-overexpressing lines was slower and more inhibited than that of Col plants when the seeds were sown in normal MS medium (**Figure 7A**). Ectopic *SiNCED1*-overexpressing line seeds sown in MS medium containing high NaCl, mannitol, and glucose showed germination delay, which revealed that *SiNCED1*-overexpressing lines were more sensitive to high salt, high mannitol, and high glucose conditions than Col plants (**Figures 7B–D**). Furthermore, when we grew *SiNCED1*-overexpressing lines and Col seeds for 7 d in normal MS media, the Col seeds all germinated, while only 87.58% and 85.60% of the seeds of *SiNCED1*-overexpressing lines OE1-3 and OE1-5 germinated, respectively (**Figures 7A**, **8A**). Sowing the seeds in the MS medium containing high NaCl, mannitol, and glucose for 7 d, the phenotype of *SiNCED1*-overexpressing lines OE1-3 and OE1-5 showed that the seeds could not germinate as well as those of Col (**Figures 8B–D**).

## 4 Discussion

ABA is a key hormone in plants that regulates plant development, including seed germination, seedling growth, stomatal aperture, flowering, and senescence. Additionally, it also elevates the ability of plants to withstand multiple stresses (Finkelstein, 2013; Kumar et al., 2022). NCED is the key rate-limiting enzyme in ABA biosynthesis that modulates endogenous ABA homeostasis by oxidatively cleaving 9-*cis*-violaxanthin or 9-*cis*-neoxanthin to produce xanthoxin (Priya and Siva, 2015). Currently, the *NCED* gene has been cloned in many plants, and its biological functions have been reported. However, the biological function of *NCED* in foxtail millet remains unclear. In this study, four *SiNCED* genes were found in the NCBI database and cloned, after which homologous comparison were performed. The following biological functions of *SiNCED1* were identified: seed germination regulation, stomatal closure, and drought stress tolerance by modulating endogenous ABA synthesis.

*NCED* belongs to the *CCD* gene family, which ordinarily contains the conserved REP65 domain (Yue et al., 2022; Zhao et al., 2022). In our study, the phylogenetic tree and multiple alignment analysis showed that SiNCED1 was separately grouped into one cluster, while the other three SiNCEDs were aggregated into another cluster, but SiNCED1 was homologous with other NCED proteins and contains the conserved REP65 domain (**Figures 1**, **2**). Members of the *NCED* subfamily are involved in the synthesis of ABA, which is involved in plant growth and development (Yang et al., 2022). *OsNCED2* was constitutively expressed in various tissues in rice plants; *osnced2* mutant seedlings with knockout of *OsNCED2* showed growth inhibition (Copenhaver et al., 2021). *OsNCED3* is expressed in multiple tissues, including embryo, coleoptile, root, leaf, culm, node, flower, stigma, and pollen, with especially high expression found in the flower and root. However, *nced3* mutant seedlings with a mutation in the *OsNCED3* gene showed a faster growth trend as well as longer roots and shoots compared with the wild type (Huang et al., 2018). Furthermore, *osnced2* and *nced3* mutants showed contrary seedling phenotypes. The regulation of endogenous ABA content was regarded as the main mechanism for the regulation of the growth and development of seedlings by *OsNCED2* and *OsNCED3*, which also confirms the dual regulatory effect of endogenous ABA on plant growth and development (Cheng et al., 2002). Expression pattern analysis showed that *SiNCED1* was expressed in multiple organs, especially the roots, which showed the highest expression (**Figure 3A**). However, the seedling growth and development of ectopic *SiNCED1*-overexpressing lines showed no difference from those of Col. Whether *SiNCED1* modulates the growth and development of foxtail millet needs to be investigated by constructing *nced1* mutants of foxtail millet. In addition, the transcript levels of *SiNCED1* were significantly induced by ABA, osmotic stress, and high salt stress (**Figures 3B–G**). *SiNCED1* is likely similar to other *NCEDs* in other vascular plants, such as *AtNCED3* in Arabidopsis and *OsNCED3* and *OsNCED4* in rice, which are significantly induced by high salt and osmotic stress (Huang et al., 2018; Hwang et al., 2018).

ABA is an important plant stress hormone. Under water stress, ABA can rapidly synthesize and regulate ABA-dependent signaling pathways to resist water stress, which are in turn regulated by *NCED* genes (Ye et al., 2012; Kavi Kishor et al., 2022). In our study, the ectopic overexpression of *SiNCED1* in Arabidopsis enhanced its ability to survive water stress (**Figure 4B**), and increased endogenous ABA levels under water stress (**Figure 6A**). *NCED* genes that participate in drought stress have also been reported in many plants. For instance, heterologous expression of *CrNCED1* in tobacco has indicated that this gene can enhance drought stress tolerance by increasing ABA levels and decreasing the levels of reactive oxygen species (Xian et al., 2014). Constitutively overexpressing *VaNCED1* in a drought-sensitive cultivar of *V. vinifera* increased ABA content and decreased stomatal density, thereby significantly strengthening drought resistance (He et al., 2018). *GhNCED3a/3c* participates in drought stress in cotton because *GhirNAC2* directly binds to its promoter to modulate ABA biosynthesis and stomatal closure (Shang et al., 2020). *AhNCED1*, cloned from peanuts, can control ABA content, and the negative feedback loop regulated by the transcription factor complex *AhAREB1* cooperates with *AhNAC2* under water stress (Liu et al., 2016). *AtNCED3* mediates drought stress by increasing endogenous ABA levels and promoting drought- and ABA-inducible genes expression. These signaling pathways are manipulated by NGATHA1, which binds directly to the *AtNCED3* promoter of the NBE cis-element (Sato et al., 2018). *OsNCED4* can also elevate ABA levels to increase drought tolerance through *OsbZIP23*, which is directly bound to its promoter cis-element, ABRE (Zong et al., 2016).

Under drought conditions, plants produce and accumulate ABA in their guard cells, which induces stomatal closure to reduce water loss (Lim et al., 2015; Wang et al., 2022). *NCED* genes have been identified as a major determinant of endogenous ABA levels by regulating ABA biosynthesis (Tan et al., 1997; Schwartz et al., 2003). Our results suggest that *SiNCED1* overexpression in Arabidopsis can reduce water loss by increasing the percentage of completely closed stomata and reducing stomata apertures under water stress (**Figure 5**). This is consistent with the modulation of ABA biosynthesis and stomatal closure by *GhNCED3a/3c* under drought stress (Shang et al., 2020) and *AtNCED3* expression induced by the small peptide CLE25 to enhance ABA levels and modulate stomatal movement to prevent water loss (Takahashi et al., 2018). In addition, proline content and electrolyte leakage are important physiological indices for the evaluation of plant resistance to water stress (Voetberg and Sharp, 1991; Jiang et al., 2016). The ectopic expression of *BnNCED3* in Arabidopsis or overexpression of *OsNCED5* in rice resulted in both exhibiting higher proline content and lower electrolyte leakage than the wild type following drought stress treatment (Xu and Cai, 2017; Huang et al., 2019). In the present study, *SiNCED1*-overexpressing transgenic lines had higher proline content and lower electrolyte leakage than Col under water stress (**Figures 4D, F** **)**, which is reliable evidence for the role of *SiNCED1* in resistance to drought stress. Furthermore, high expression levels of ABA-related stress-responsive marker genes, such as *AtPP2C*, *AtCOR15A*, *AtRd29B*, *AtSOS1*, and *AtLTP3*, enhance resistance to drought stress in Arabidopsis (Guo et al., 2013; Yang et al., 2018; Lu et al., 2019). We found that the expression levels of these stress-responsive genes increased substantially in *SiNCED1* transgenic lines (**Figures 6B–F**), indicating that *SiNCED1* can also help to maintain a high resistance to water stress.

ABA has an important effect on seed dormancy, which relies on its biosynthesis and catabolism (Bentsink and Koornneef, 2008). Numerous studies have showed that the overexpression of ABA biosynthesis pathway genes, including *PtNCED1* (Lee et al., 2018), *AtNCED9* (Seo et al., 2016), *BdNCED1* (Barrero et al., 2012), *TaNCED2* (Izydorczyk et al., 2018), and *LeNCED1* (Tung et al., 2008), can promote seed dormancy by regulating endogenous ABA accumulation. In the present study, *SiNCED1* overexpression in transgenic lines exhibited the inhibition of seed germination under normal and abiotic stress conditions (**Figures 7** and **8**), which showed a similar phenotype to that in the above-mentioned reports. However, the molecular mechanism underlying the regulation of dormancy and germination processes in foxtail millet seeds by *SiNCED1* still requires further study.

In the present study, four *NCED* genes were identified and cloned into foxtail millet. The relative expression levels of *SiNCED1* were strongly induced in the shoots and roots under osmotic stress. Overexpression of *SiNCED* confers drought stress tolerance to Arabidopsis. This function is supported by the finding that *SiNCED1* regulates ABA biosynthesis to modulate stress-related physiological indices, stomatal closure, and ABA-related stress-responsive genes. Furthermore, *SiNCED1* promotes seed dormancy by increasing ABA accumulation. Thus, *SiNCED1* is likely crucial for improving the breeding of foxtail millet. The mechanisms by which *SiNCED1* cooperates with other *SiNCED* members and how transcription factors regulate its expression and affect developmental processes and abiotic stress could be novel research topics for future studies.

## Data availability statement

The original contributions presented in the study are included in the article/**Supplementary Material**, further inquiries can be directed to the corresponding author.

## Author contributions

Conceptualization, ML and LC. Formal analysis, SY and FW. Investigation, YH and YJ. Supervision, ML and LC. Writing—original draft, YH and SY. Writing—review and editing, ML and DM. All authors contributed to the article and approved the submitted version.
