# Supplementary material for: SiNCED1, a 9-cis-epoxycarotenoid dioxygenase gene in Setaria italica, is involved in drought tolerance and seed germination in transgenic Arabidopsis
Source: Front Plant Sci. 2023 Mar 9;14:1121809. doi: 10.3389/fpls.2023.1121809 (PMC10034083; doi:10.3389/fpls.2023.1121809)
Supplement: Supplementary file 1 [file Table_1.docx]

**Table S1. Primers and oligos used in this study**

| Name | Primer sequence (5’-3’) |
| --- | --- |
| **Plasmid Constructions** | |
| SiNCED1-F | GACAAGGATCAACCACCAGTTC |
| SiNCED1-R | ACTCACGAAACCCTTTTATGG |
| pHB-SiNCED1-F | CCCAAGCTTATGGAGAGAACACTGATCACCT |
| pHB-SiNCED3-R | GCTCTAGATCAGTTTGTGTGTGTTTTCTCTT |
| **qRT-PCR** | |
| qSiNCED1-F | CGCATCCTTCAAGGTCGTCTGG |
| qSiNCED1-R | GCTCTTGCGGTAGCGGTTCTT |
| SiActin-F | GGCAAACAGGGAGAAGATGA |
| SiActin-R | GAGGTTGTCGGTAAGGTCACG |
| AtActin-F | CATCAGGAAGGACTTGTACGG |
| AtActin-R | GATGGACCTGACTCGTCATAC |
| AtPP2C-F | AAGCCGTATGTAATCAGCA |
| AtPP2C-R | CAAGCCTCGTCAGCAACAG |
| AtCOR15A-F | GAGGCCACAAAGAAAGCTTC |
| AtCOR15A-R | CTTTGTGGCATCCTTAGCCTC |
| AtRd29B-F | GAGTGAAGGAGACGCAACAAGGG |
| AtRd29B-R | GGTTTACCACCGAGCCAAGAAGT |
| AtSOS1-F | TCATCATCCTCACAATGGCTCTAA |
| AtSOS1-R | ACCAACTTGCGTGGGACAACTTTA |
| AtLTP3-F | GGCTTTCGCTTTGAGGTTCTTC |
| AtLTP3-R | ATGCTAACACCGCACTTTCCAG |
