# Supplementary material for: SiNCED1, a 9-cis-epoxycarotenoid dioxygenase gene in Setaria italica, is involved in drought tolerance and seed germination in transgenic Arabidopsis
Source: Front Plant Sci. 2023 Mar 9;14:1121809. doi: 10.3389/fpls.2023.1121809 (PMC10034083; doi:10.3389/fpls.2023.1121809)
Supplement: Supplementary file 2 [file Table_2.doc]

**Table S2. The CDS sequence of four *SiNCED* genes**

| **Gene name** | **Sequence** |
| --- | --- |
| *SiNCED1*  (LOC101783411, 1980 bp) | ATGGAGAGAACACTGATCACCTCTAACCTCTCCATGGCTGCGCATCCTTCAAGGTCGTCTGGGAGAGTTCACTACATCTCCCCTGCAGCCTCTGCTGCTGCACAGAACTCCAGCTACAACAGGAAGAAGAGCACCCCGTCCCGACCACCGCCGTCGGCCGCCGCAACCGCCACCGTCGTCACCTCCCCTCCTCCAGCCACTGACAATGCTCAGCCCGCCGCTCCGAAGCAGGCCGAGCAGCAGGAGAAGGGAGAGCGCGTGGCGGCCAAGACCACCACTTCAAGAACCGCTACCGCAAGAGCAAGGGCGCCGACGAGCCAGGCTCTGGCCCGGCCTCGCCGCCGCCCTGCCCCGGCGGCCGCGTCCCTGCCGATGGCGTTCTGCAGCGCGCTGGAGGAGGCGATCAACACGTTCGTGGACCCGCCGGCGCTGCGGCCGTCGGTGGACCCGCGGAACGTGCTGTCTGCCAACTGGGCTCCCGTCGACGAGCTGCCGCCGACGCCCTGCCCCGTCGTGCGCGGCGTCATCCCACGCTGCCTCGCCGGTGGGGCCTACATCCGCAACGGGCCCAACCCGCAGCACCTCCCGCGCGGGCCGCACCACCTCTTCGACGGCGACGGCATGCTGCACTCCCTGCTCCTCCCGGCGGCGGACTCGCCGCCGTCGGCCGACCCCATTCTGTGCTCGCGGTACGTGCAGACGTACAAGTACATCGTGGAGCGCGACGCCGGCGCGCCGGTGATGCCGAACGTCTTCTCCGGCTTCCACGGCCTGGCCGGGTTCGCGCGCGGCGCCGTCGTGGCGGCAAGGGTGCTGACGGGGCAGATGAACCCGGCCGAGGGCGTGGGGCTCGCCAACACCAGCCTCGCCTTCTTCGGCGGGCGCCTCTACGCGCTGGGCGAGTCAGACCTCCCCTACGCCGTGCGCGTCGACCCGGCCACCGGCGAGGTGACCACGCACGGCCGGTGCGACTTCGGCGGCCGCCTCTTCATGGGCATGACCGCGCACCCCAAGAAGGACCCCATCACCGGCGAGGTCTTCGCGTTCCGCTACGGGCCCGTCCCCCCGTTCGTCACCTACTTCCGGTTCGACGCCGCCGGGAACAAGGGCCCCGACGTGCCCATCTTCTCCGTGATGCAGCCGTCGTTCCTGCACGACTTCGCCGTCACCGAGCGTTACGCCATCTTCCCGGAGATCCAGATCGTGATGCAGCCCATGGGCATGGTGGCCGGCGGCGCGCCCGTCGGATCGGACTCCGGCAAGGTGCCCCGGATCGGCGTGCTCCCCAAGTACGCCACGGACGAGTCGGAGATGCGGTGGTTCGAGGTGCCGGGCTTCAATATCATGCACACGCTGAACGCGTGGGAGGAGGCCGGCGGCGACGAGCTGGTGCTGGTGGCGCCCAACGTCCTGTCCGTGGAGCACGCGCTGGAGCGCATGGAGCTCGTGCACGGCTGCGTCGAGAAGGTGCGCATCGACCTGCGCACGGGCGCCGTGTCGCGCACCCCGCTCTCGGCGGGGAACCTCGACTTCGGCGTCATCCACCCGGGCTACCTCGGCCGGCGCAACCGGTACGGCTACTTCGGGATCGGCGACCCCATGCCCAAGATCAGCGGGGTGGCGAAGCTGGACCTGGAGCGCGCCGGCACTGGAGACTGCACCGTGGCGACGCGGGACTTCGGGCCCGGGTGCTTCGCCGGGGAGCCCTTCTTCGTGCCCGACGACGTGGAGGGGGACGGCAACGAGGACGACGGCTACGTGGTGTGCTATGTCCACGACGAGCGCACGGGGGAGAACAGGTTCGTGGTGATGGACGCGCGGTCGCCGACGCTGGACATCGTCGCCGAGGTGCAGCTGCCCGCACGCGTCCCCTACGGCTTCCACGGCCTCTTCGTCACGCAGGCCGAGCTCCAGGCGCAGCAGCGATGGTCGGAGCATACCAGGAGCACAGAATTCCAAGAGAAAACACACACAAACTGA |
| *SiNCED3*  (LOC101778945, 1890 bp) | ATGGCGATGCTTGTTCGGGGTCTCGCTCCGCCGCCCACCTCTGTTTCTTCCATACACCGGCGCCTGCCGGCCGGGTCAAGGGCCCGGGGCCCCAATTCGGTGAGGTTCTCGCCGCGCGCGGTCAGCTCCGTGCCCGCCGATTGCCTGCCGGCGGCGGCGTTCAAGCCCACCGAGCTGACGGTGCCCAAGAAGCCCGCCGCCATTGCCGCGCCGCCGAGGCCCACGGCGGCGCCGGCCTCAACGCCGCGGAAATCGGCGGGGAAGAAGAAGGAGCTCAACCCCTTCCAGCGCGCCGCGGCGGCGGCGCTCGATAAGTTCGAGGAGGCGTTCGTGGCGGGCGTCCTCGAGCGCCCCCACGGCCTGCCCCGGACGGCCGACCCGGCCGTGCAGATCGCCGGCAATTTCGCGCCCGTCGGGGAGAGGCCCCCCACCCGCGAGCTCCCGGTCACCGGCCGCATCCCGCCCTTCATCAACGGCGTGTACGCGCGCAACGGCGCCAACCCCTCCTTCGACCCCGTCGCCGGGCACCACCTCTTCGACGGCGACGGCATGGTGCACGCGCTCCGGATACGGAACGGCGCCGCCGAGTCCTACGCGTGCCGCTTCACCGAGACCGCGCGCCTGCGCCAGGAGCTCGCGATCGGCAGGCCCGTCTTCCCAAAGGCCATTGGCGAGCTGCACGGTCACTCCGGGATCGCGCGCCTTGCCCTGTTCTACGCGCGCGCCGCGTGCGGTCTCGTCGATCCCTCCCACGGCACCGGCGTCGCCAACGCCGGCCTTGTCTACTTCAACGGCCACCTCCTCGCCATGTCCGAGGACGACCTCCCGTACCACGTGCGCGTCACGGACGGCGGCGACCTCGAGACCGTCGGCCGCTACGACTTCGACGGCCAGCTCGGCTGCGCCATGATCGCGCACCCCAAGCTCGACCCGGCCACCGGCGAGCTCCACGCGCTCAGCTACGACGTCATCAAGAAGCCGTACCTCAGGTACTTCTACTTCAGGCCCGACGGCACCAAGTCCGACGACGTCGAGATCCCGCTCGACCAGCCGACCATGATCCACGACTTCGCCATCACCGAGAACTTCG TCGTCGTGCC CGACCACCAG GTGGTGTTCA AGCTCCAGGA GATGCTGCGCGGCGGCTCGCCCGTGGTGCTGGACAAGGAGAAGACCTCGCGCTTCGGCGTGCTCCCCAAGCACGCCAAGGACGCGTCGGAGATGGCGTGGGTGGACGTGCCGGACTGCTTCTGCTTCCACCTCTGGAACGCGTGGGAGGACGAGGAGACCGGCGAGATCGTCGTCATCGGCTCCTGCATGACCCCCGCCGACTCCATCTTCAACGAGTCCGACGAGCACCTCGAGAGCGTGCTCACGGAGATCCGCCTCGACACCCGCACCGGCCGCTCCACGCGGCGCGCTATCCTGCCGCCGTCGCAGCAGGTGAACCTGGAGGTGGGCATGGTGAACCGCAACCTCCTGGGCCGCAAGACTCGGTACGCGTACCTCGCCGTGGCCGAGCCGTGGCCCAAGGTGTCGGGCTTCGCCAAGGTGGACCTGGCCACCGGCGAGATGACCAAGTTCGAGTACGGCGATGGCCGGTTCGGCGGCGAGCCCTGCTTCGTGCCCATGGACCCCGCCGCCGCCCACCCGCGCGGCGAGGACGACGGGTACGTGCTCACCTTCGTGCACGACGAGCGCGCCGGCACGTCGGAGCTCCTCGTCGTCAATGCCGCCGACATGCGGCTGGAGGCCACGGTCCAGCTCCCGTCCCGCGTGCCCTTCGGCTTCCACGGCACCTTCATCACGGCCAACGAGCTCGGAGGCCCAAGGCCTGATCGCCCGAGCTCCCGTTCGGTTCCCCACCTACCTCCACGTGAGGACTCGTAG |
| *SiNCED4*  (LOC101766978, 1749 bp) | ATGGCGTCGTCCGTCACCGCTCCCCCGGCGGCGGCTCCGGCCACCGCGCCGGCGAAGCCCAAGAAGCCGTCGCAGCTCAAACCGAGCACCGGGACCGGCAAGGCCTCCCGCACCGCGGCCCCCGTGCGGCCAATGCGCGCCGCCACCCCGGCCGGCCCCAAGTGGAACCCGTTCCAGCGGCTCGCGGCGGCGGCGCTGGACGCCGTCGAGGACGGCCTCGTCGCGGGCCTCCTCGAGCGCGCGCACCCGCTCCCCCGCACCGCCGACCCCGCCGTCCAGATCGCCGGCAACTACGCGCCCGTCGGGGAGCGCCCGCCCCGCCGCCGCGGCGAGGCGGCGCCGCGCCCGCTCCCCGTCTCCGGCCGCGTCCCGCCCTGCCTCGACGGGGTCTACGTCCGCAACGGCGCGAACCCGCTCCACGAGCCCCGCGCGGGGCACCACCTCTTCGACGGCGACGGGATGCTCCACGCCGTGCGCCTCCGCGCCGGGCGCGCCGAGTCCTACGCGTGCCGGTTCACGGAGACGGCGCGGCTCCGGCAGGAGCGCGCCATCGGCCGCGCGGTATTTCCAAAGGCCATCGGCGAGCTCCACGGCCACTCCGGCGTCGCCCGCCTCCTCCTCTTCGGCGCCCGCTCCCTCTGCGGCGTCCTCGACGCGTCCCAGGGGATCGGGGTTGCCAACGCCGGCCTCGTGTTCCATAACAACCGCCTCCTCGCCATGTCCGAGGACGACCTCCCGTACCACGTCCGCGTCACCGCCGACGGCGACCTCGAGACCGTCGGTCGCTACGACTTCGGCGGCCAGCTCGAAGGCGCCATGATCGCGCACCCCAAGCTCGACCCGGCCACCGGCGAGCTCTTCGCGCTCAGCTACAATGTCGTCTCCAAGCCGTACCTCAAGTACTTCTACTTCACCGCTGACGGCCGCAAGTCCCCCGACGTCGAGATCCCCGTCGACGCGCCCACCATGATGCACGACTTCGCCGTCACCGAGAACTACGCCATCATCCCCGACCAGCAGATCGTCTTCAAGCTCCAGGAGATGGTTCTCGGAGGCTCCCCCGTCGTGTACGACAAGACCAAGACCTCGCGGTTCGGGGTGCTCCCGAAGCGCGCCGCCGACGCGTCGGAGCTCCGGTGGGTGGAGGTCCCCGACTGCTTCTGCTTCCACCTCTGGAACGCGTGGGAGGACGACGCTACCGGCGAGATCGTCGTGATCGGGTCCTGCATGACCCCGGCCGACGCCGTCTTCAACGAGTCCGGCGAGGGGGAGGCCTTCCAGAGCGTGCTGTCGGAGATTCGCCTCGACCCCACCACCGGCACGTCGCGGCGGCGCGCTGTGCTGGCCGCCGACGACCAGGTGAACCTCGAGGCCGGGATGGTGAACCGGCAGCTGCTGGGCCGCAAGACCCGGTACGTCTATCTCGCCATCGCCGAGCCGTGGCCCAAGGTGTCGGGGTTCGCCAAGGTGGACCTGGAGACCGGCACCGCCGAGAAGTTCATCTACGGCGAGGGCCGGTACGGCGGCGAGCCCTGCTTCGTGCCGCGCCCGGGCGCCGGCGCCGGCGCGGAGGAGGACGACGGCTACGTGCTTTGCTACGTCCACGACGAGGCCCGCGGCGCGTCGGAGATGCTCGTCGTCAACGCCCGCGACATGCGGGAGGAGGCCGCCGTCAAGCTGCCGGGCCGCGTCCCGTACGGGCTGCACGGCACCTTCATCTCCGGCGAGGACCTGCAGCGGCAGGCCTAG |
| *SiNCED5*  (LOC101770668, 1809 bp) | ATGCAGACTCTCACAGCAAGCTCCCCTGCTTCTACCTCCTCCATACACCGCGGCGGCGGGTCGAGGACTCCGCGCAGCTCGGCGCGGTTCGCGCCCCGGGCCGCGGCGGCCGCGGCCACGAACTCGGTCCTCAGCGCGCCGCCCGCCGTGCGCTACGTTCCGCCTCCGAAGCCCGTCCCCAATGCCGCCGCCCCCGCGCCGGCGCGCCGCGGCGACTCGAACCACCACAGGGCGGGCGACGAGAAAGGCTTCAACTTCCTGCAGCGCGCGGCGGCGGCGGCGCTGGACGCGTTCGAGGCCGGCGTCATCACCAACCTCCTCGAGAGGCCGCGCGCGCTGCCGCGGGCGGCGGACCCCGCCGTGCAGATCGCCGGCAACTTCGCGCCGGTCGGGGAGCAGCCGCCCGTGCGTGCGCTCCCGGTCTCCGGACGCATCCCGCCCTTCATCAACGGCGTGTACGCGCGCAACGGCGCCAACCCGTGCTTCGAACCCACCGCCGGGCACCACCTCTTCGACGGCGACGGCATGGTGCACGCCGTCCGCATCCGGAACGGCGCCGCGGAGTCGTACGCTTGCCGGTTCACGGAGACGGCGAGGCTCCGGCAGGAGCGCGCGCTGGGGCGGGCGGTGTTCCCCAAGGCCATCGGCGAGCTCCACGGCCACTCCGGGATCGCCCGCCTCGCCCTCTTCTACGCGCGCGGCCTCTGCGGCCTCGTCGACCCCTCCCACGGCACCGGCGTCGCCAACGCCGGCCTCGTCTACTTCAACGGCCGCCTCCTCGCCATGTCCGAGGACGACCTCCCGTACCAGGTCCGCGTCACCGGCGACGGCGACCTCGAGACGGTCGGGCGGTACGACTTCGACGGCCAGCTCGGGTGCGCCATGATCGCGCACCCCAAGCTCGACCCGGCCTCCGGCGAGCTCTTCGCGCTCAGCTACGACGTGATCAAGCGCCCCTACCTCAAGTACTTCTACTTCCGCCCCGACGGCACCAAGTCCGACGACGTCGAGATCCCGCTCGACCAGCCGACCATGATCCACGACTTCGCCATCACCGAGAACTTCGTCGTGGTTCCCGACCACCAGGTGGTGTTCAAGCTCGGCGAGATGTTCCGCGGCGGCTCACCCGTGGTCCTCGACGGCGAGAAGACCTCCCGCTTCGGCGTGCTGCCCAAGTACGCCGGCGACGCGTCGGAGATGGTGTGGGTGGACGTGCCGGACTGCTTCTGCTTCCACCTCTGGAACGCGTGGGAGGAGCCGGAGTCCGACGAGGTCGTCGTCATCGGCTCCTGCATGACCCCCGCCGACTCCATCTTCAACGACTCCGGCGACGACCGCCTCGAGAGCGTGCTCACGGAGATCCGCCTCAACACCCGCACCGGCGCCTCCACGCGCCGCGCCGTGCTGCCGCCGGCGAGCCAGGTGAACCTGGAGGTGGGCATGGTGAACCGGAACATGCTGGGGAGGAAGACGCGGTACGCGTACCTCGCCGTGGCGGAGCCGTGGCCCAAGGTGTCCGGGTTCGCCAAGGTCGACCTCGCCACCGGCGAGCTCACCAGGTTCGAGTACGGCGAGGGGCGGTTCGGCGGCGAGCCCTGCTTCGTGCCGACCGAGGGCGCGCCGGCACGCGGCGAGGACGACGGCTACATCCTCTCCCTGGTCCGCGACGAGCGCGCAGGGACGTCGGAGCTCCTGGTGGTGAACGCCGCAGACATGCGGCTCGAGGCCACCGTCCAGCTCCCCTCCCGCGTCCCCTACGGCTTCCATGGCACCTTCATCGGCGCCAAGGAGCTGGAAGCCCAAGCCTGA |
