# Supplementary material for: SiNCED1, a 9-cis-epoxycarotenoid dioxygenase gene in Setaria italica, is involved in drought tolerance and seed germination in transgenic Arabidopsis
Source: Front Plant Sci. 2023 Mar 9;14:1121809. doi: 10.3389/fpls.2023.1121809 (PMC10034083; doi:10.3389/fpls.2023.1121809)
Supplement: Supplementary file 3 [file Table_3.doc]

**Table S3. Functional prediction of *cis*-acting elements of *SiNCED1* promoter**

| ***Cis*-element** | **Core sequence** | **Function** |
| --- | --- | --- |
| A-box | CCGTCC | *cis*-acting regulatory element |
| ABRE | ACGTG | *cis*-acting element involved in the abscisic acid responsiveness |
| ARE | AAACCA | *cis*-acting regulatory element essential for the anaerobic induction |
| CAAT-box | CCAAT | common *cis*-acting element in promoter and enhancer regions |
| CAT-box | GCCACT | *cis*-acting regulatory element related to meristem expression |
| CGTCA-motif | CGTCA | *cis*-acting regulatory element involved in the MeJA-responsiveness |
| G-box | CACGTC | *cis*-acting regulatory element involved in light responsiveness |
| GA-motif | ATAGATAA | part of a light responsive element |
| GATA-motif | GATAGGG | part of a light responsive element |
| GCN4-motif | TGAGTCA | *cis*-regulatory element involved in endosperm expression |
| L-box | ATCCCACCTAC | part of a light responsive element |
| LAMP-element | CTTTATCA | part of a light responsive element |
| MBS | CAACTG | MYB binding site involved in drought-inducibility |
| MRE | AACCTAA | MYB binding site involved in light responsiveness |
| O2-site | GATGATGTGG | *cis*-acting regulatory element involved in zein metabolism regulation |
| P-box | CCTTTTG | gibberellin-responsive element |
| TATA-box | TATA | core promoter element around -30 of transcription start |
| TC-rich repeats | GTTTTCTTAC | *cis*-acting elemant involved in defense and stress responsiveness |
| TCT-motif | TCTTAC | part of a light responsive element |
| TGACG-motif | TGACG | *cis*-acting regulatory element involved in the MeJA-responsiveness |
